# MLW-gcForest: a multi-weighted gcForest model towards the staging of lung adenocarcinoma based on multi-modal genetic data

**DOI:** 10.1186/s12859-019-3172-z

**Published:** 2019-11-14

**Authors:** Yunyun Dong, Wenkai Yang, Jiawen Wang, Juanjuan Zhao, Yan Qiang, Zijuan Zhao, Ntikurako Guy Fernand Kazihise, Yanfen Cui, Xiaotong Yang, Siyuan Liu

**Affiliations:** 1 College of Information and Computer, Taiyuan University of Technology, Taiyuan, 030024 China; 2 College of Information Technology and Engineering, Jinzhong University, Jinzhong, 030619 China; 3 Department of Radiology, Shanxi Province Cancer Hospital, Taiyuan, 030013 China; 4 College of Computer Engineering and Science, Shanghai University, Shanghai, 200444 China

**Keywords:** MLW-gcForest staging model multi-modal genetic data lung adenocarcinoma

## Abstract

**Background:**

Lung cancer is one of the most common types of cancer, among which lung adenocarcinoma accounts for the largest proportion. Currently, accurate staging is a prerequisite for effective diagnosis and treatment of lung adenocarcinoma. Previous research has used mainly single-modal data, such as gene expression data, for classification and prediction. Integrating multi-modal genetic data (gene expression RNA-seq, methylation data and copy number variation) from the same patient provides the possibility of using multi-modal genetic data for cancer prediction. A new machine learning method called gcForest has recently been proposed. This method has been proven to be suitable for classification in some fields. However, the model may face challenges when applied to small samples and high-dimensional genetic data.

**Results:**

In this paper, we propose a multi-weighted gcForest algorithm (MLW-gcForest) to construct a lung adenocarcinoma staging model using multi-modal genetic data. The new algorithm is based on the standard gcForest algorithm. First, different weights are assigned to different random forests according to the classification performance of these forests in the standard gcForest model. Second, because the feature vectors generated under different scanning granularities have a diverse influence on the final classification result, the feature vectors are given weights according to the proposed sorting optimization algorithm. Then, we train three MLW-gcForest models based on three single-modal datasets (gene expression RNA-seq, methylation data, and copy number variation) and then perform decision fusion to stage lung adenocarcinoma. Experimental results suggest that the MLW-gcForest model is superior to the standard gcForest model in constructing a staging model of lung adenocarcinoma and is better than the traditional classification methods. The accuracy, precision, recall, and AUC reached 0.908, 0.896, 0.882, and 0.96, respectively.

**Conclusions:**

The MLW-gcForest model has great potential in lung adenocarcinoma staging, which is helpful for the diagnosis and personalized treatment of lung adenocarcinoma. The results suggest that the MLW-gcForest algorithm is effective on multi-modal genetic data, which consist of small samples and are high dimensional.

## Introduction

Lung cancer is one of the most common cancers and possesses the highest morbidity and mortality, causing more than 1.4 million deaths each year. Lung cancer can be classified into non-small cell lung cancer (NSCLC) and small cell carcinoma. Lung adenocarcinoma and lung squamous cell carcinoma are common types of NSCLC [1, 2], with lung adenocarcinoma accounting for approximately 70% of NSCLC. Therefore, the study of lung adenocarcinoma is crucial in the study of lung cancer. The 5-year survival rate of lung adenocarcinoma does not exceed 5% [3]. Different treatments are needed during different stages of lung adenocarcinoma to improve the patient’s survival rate. Therefore, the accurate staging of lung adenocarcinoma is the first step in clinical diagnosis and targeted treatment.

With the development of high-throughput sequencing technology, a large number of microarrays and genetic data have been produced. An increasing number of researchers have engaged in the analysis of genetic data. As an important branch of artificial intelligence, machine learning methods are favored by many researchers. Various machine learning methods based on cancer gene data have been widely used in disease prognosis and prediction. Based on the Cancer Genome Atlas (TCGA) and Stanford Tissue Microarray Database, Yu et al. [4] used regularized machine learning methods to select the top quantitative image features and classified patients as having lung adenocarcinoma and squamous cell carcinoma. Cai et al. [5] used machine learning methods to capture unbiased and compact molecular features to classify lung adenocarcinoma, small cell lung cancer, and NSCLC. Li et al. [6] proposed a method that combines support vector machine (SVM) and random forest to predict lung cancer adenocarcinoma stages. Nguyen et al. [7] proposed a multi-class machine learning technique using SVM to classify tumor node metastasis (TNM) staging of lung cancer patients by analyzing their free-text histology reports. Singh et al. [8] used machine learning methods to identify biomarkers and constructed a model to distinguish early and late stages of papillary renal cell carcinoma based on gene expression profiles. Xiao et al. [9] used an ensemble deep neural method comprising five machine learning models to predict cancer. The proposed deep learning-based multi-modal ensemble method achieved better predictive performance than that of any single model. Many machine learning or deep learning algorithms classify or predict cancer by analyzing different types of cancer gene data [10–22]. Many scholars have analyzed and studied lung cancer gene data using other methods [23–27]; however, to the best of our knowledge, few studies have applied machine learning to the staging of lung adenocarcinoma based on multi-modal genetic data.

Multi-modal genetic data mainly include gene expression RNAseq (RNA-seq), methylation data, and copy number variation (CNV) from the same patients, which are usually characterized by small sample sizes and high dimensionality. The construction of deep neural networks typically relies on a large amount of data: small samples and high-dimensional genetic data increase the risk of overfitting deep neural networks during training. A deep forest model called gcForest [28] was recently proposed as an alternative method of deep learning to alleviate the overfitting problem of deep neural networks for small samples.

The gcForest model is a new decision tree integration of the deep forest method. The algorithm is a combination of traditional machine learning algorithms and deep learning ideas. The gcForest model implements multi-grained scanning to further enhance the learning ability and can achieve good performance in high-dimensional, small-scale data. The gcForest model adopts a cascade structure in which each layer receives information processed by the previous layer and transmits information to the next layer. The standard gcForest algorithm is expected to deliver better predictions than those of traditional machine learning methods, even in cases of small-scale training data [28]. However, the gcForest algorithm still has the following shortcomings in the analysis of cancer genetic data: (1) the multi-grained scanning of the gcForest algorithm does not account for the different effects of each random forest on the final prediction, which is not conducive to capturing diverse features, especially in small-sample data; and (2) the class vectors obtained under different scanning granularities have different effects on the final classification decision-making ability, but the standard gcForest algorithm simply concatenates the class vectors from different granularity sliding windows, which potentially weakens the final classification ability.

In our previous work, an improved gcForest algorithm based on methylation data [29] was proposed and successfully applied to the subtype classification of cancer. In this paper, we have substantially revised and applied to the staging model of lung adenocarcinoma.

The main contribution of our approach is the proposal of MLW-gcForest to construct a staging model of lung adenocarcinoma based on multi-modal genetic data. Specifically, (1) we set dynamic weights for different random forests in the multi-grained scanning according to the classification performance of each random forest; (2) we propose a sorting optimization algorithm to set different weights for each sliding window, as the class vectors generated by each sliding window have varying effects on the final prediction results; and (3) we adopt decision-level fusion to construct a staging model of lung adenocarcinoma based on multi-modal genetic data.

## Method

### Feature selection

In our experiment, lasso regression is used for feature selection [30]. The lasso method belongs to a class of embedded feature selection methods that can overcome the problems of efficiency and computational cost in traditional feature selection and has been successfully applied to microarray classification and gene selection [31].

The lasso method uses a paradigm penalty-based regression to find the optimal solution for formulas (1) and (2).

1 $$ \arg \underset{\updelta}{\min}\left\{\sum \limits_{i=1}^n{\left({y}_i-{\updelta}_0-\sum \limits_{j=1}^r{x}_{ij}{\updelta}_j\right)}^2\right\} $$

2 $$ \mathrm{subject}\ \mathrm{to}\sum \limits_{j=1}^r\mid {\updelta}_j\mid \le z $$

where r is the number of features of the data, n is the number of samples, δ_*j*_ is the regression coefficient of the *g* th variable, and *z* is the constraint value, which is a paradigm penalty for the regression coefficient δ_*j*_. The value of *z* can vary from 0 to infinity. When *z* is small, some variable coefficients with small effects are compressed to 0 so that these variables are deleted to achieve feature selection. When *z* is sufficiently large, it no longer constitutes an actual constraint, and all the attributes are selected.

### GcForest

**Fig. 1:**
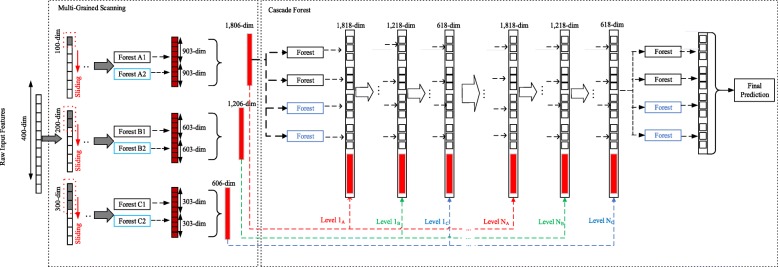
Illustration of the cascade forest structur e[28]

The gcForest model comprises two components [28], as shown in Fig. 1. (1) The first component is multi-grained scanning, which adapts sliding windows to cut raw features into feature vectors. After feeding these feature vectors into different types of random forest, the model outputs class vectors. Then, all the class vectors are concatenated and output as the result of the multi-grained scanning. (2) The second component is the cascade forest. Each cascade layer is composed of multiple random forests, which comprise decision trees. The input is composed of the class vectors from the output of the first component. Each cascade layer outputs a new class vector that is concatenated with the original class vector to form a new class vector as the input of the next layer (detail in [28]). With multiple random forests in each cascade, more discriminative features can be learned from the input vector to that cascade. A more accurate prediction is finally obtained through the layer-by-layer transfer of each cascade layer. k-fold cross-validation is used to reduce the risk of overfitting when extending a new layer. Specifically, the training data are divided into k folds. k-1 folds are selected as the training data in turn, and the remaining fold is used as the validation data. After extending the new layer, the performance of the entire cascade is estimated on the validation data, and if no significant performance gain is observed, the training process is terminated. Finally, the average of each class probability is calculated from the class vectors of the last cascade layer’s outputs: the class with the maximum probability value is used as the prediction result.

Figure 1 shows the standard gcForest model [28], which is composed of multi-grained scanning and a cascade forest. Assume that the input feature vector has 400 dimensions and that three sizes of sliding windows (100, 200, 300) are used to cut the input feature vector. The first sliding window size is 100, and the sliding stride is 1. Thus, a total of 301 scans are required, and 301*100-dimensional(dim) feature vectors are generated. These feature vectors are the input to a completely random forest and a random forest. Suppose the samples have three classes; each sample is trained using completely random forest and random forest, and 1806-dim (2*301*3 dim) class vectors are output. Similarly, when the sliding window size is 200 and 300, respectively, we obtain class vectors of 1206-dim (2*201*3 dim) and 606-dim (2*101*3 dim).

The second component is the cascade structure. The class vector output by the first component is used as input to the cascading forest component. First, the 1806-dim class vector obtained from the 100-dim sliding window is used as the input to train the first cascade layer. Notably, the diversity of forests plays an important role in constructing the model. After training four forests (two random forests [32] and two completely random forests [33]), a 12-dim class vector (4 forests, 3 classes) is generated. The 12-dim class vector is then concatenated with the original 1806-dim class vector (as shown in Fig. 1) to obtain an 1818-dim vector as the input of the second cascade layer. Similarly, the second cascade layer’s random forests are trained to generate a 12-dim class vector, which is concatenated with the class vector (1206-dim) obtained from the 200-dim sliding window in the first component. Therefore, we obtain a 1218-dim class vector as the input of the third cascade layer. Similarly, we train the third cascade layer’s random forest and obtain a 12-dim class vector, which is concatenated with the class vectors (606 dim) from the 300-dim sliding window in the first component. Therefore, we obtain a 618-dim class vector as the input of the next cascade layer. We repeat the above process each time a new layer is generated. The performance of the entire cascade is estimated on the validation set whenever a new layer is extended: if no significant performance gain is observed, the training process is terminated [28].

### Multi-weighted gcForest (MLW-gcForest)

**Fig. 2:**
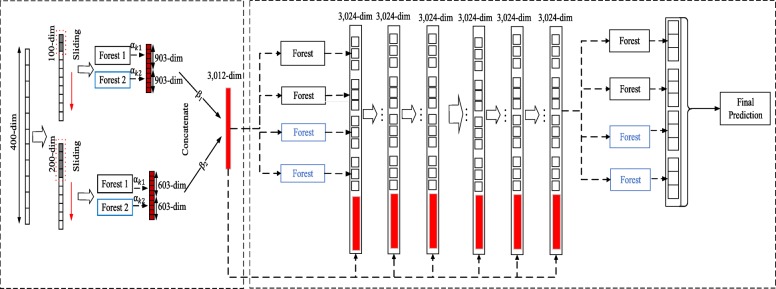
Illustration of the MLW-gcForest (multi-weighted gcForest) model. The MLW-gcForest model is composed of multi-grained scanning and a cascade forest. We made two improvements in the multi-grained scanning module: we assign different weights to the random forests according to the classification performance of each random forest and name the weights α; and we assign corresponding weights to different sliding windows and name the weights β

Two challenges may limit gcForest’s application to small-scale genetic data. 1) Each forest in the original gcForest has the same impact on the final prediction, but in reality, the classification ability of each forest is different. Therefore, we assign different weights αto the random forests according to the classification performance of each random forest. 2) In the original gcForest, for the same raw data, scanning with multiple scale windows can generate different-dimension feature vectors. Multi-grained scanning enriches the diversity of generated features. The above method of generating features can capture more comprehensive features, and the different grains of feature vectors generated under different sliding windows have different effects on the final classification results. We believe giving equal attention to different sliding windows in the original gcForest algorithm is unreasonable; therefore, we consider assigning corresponding weights β to different sliding windows. A structure diagram of MLW-gcForest structure is shown in Fig. 2.

#### Determination of weights

The weight assignment is the first improvement to the algorithm. We use one random forest and one completely random forest for each sliding window and compute the weights of each forest, α_1_ and α_2_. The specific method is as follows.

The performance of each random forest must be evaluated objectively; therefore, evaluation criteria must be introduced. The receiver operating characteristics (ROC) [34] curve is a common indicator to measure the performance of a model and indicate the performance of a classifier. In the case of a binary classification task, the area under the curve (AUC) is shown in formula (3).

3 $$ \mathrm{AUC}={\int}_0^1 ROC(u)\; du\;\mathrm{u}\in \left[0,1\right] $$

However, this metric is not intuitive for multi-class tasks because the AUC is usually used to measure the classification ability of binary classification tasks.

For multi-class tasks, Scurfiled et al. [35] proposed the concept of multiple ROC [36] analysis and the measurement of hypervolume under multi-flow (HUM) to evaluate the identification ability of the corresponding biomarkers. The classification of lung adenocarcinoma is a three-class task, so a double integral is used, as shown in formula (4).

4 $$ HUM={\int}_0^1{\int}_0^1 ROC(u){du}_1{du}_2 $$

In this paper, we use one random forest and one completely random forest to obtain *HUM*_1_ and *HUM*_2_ as the evaluation indicators for the classifier; the HUM values are then normalized to calculate the weight of each forest, as shown in formulas (5) and (6).

5 $$ {\upalpha}_1=\frac{HUM_1}{HUM_1+{HUM}_2} $$

6 $$ {\upalpha}_2=\frac{HUM_2}{HUM_1+{HUM}_2} $$

The values α_1_ and α_2_ are then used to assign weights to the class vectors produced by the different forests.

#### Sorting optimization algorithm

**Fig. 3:**
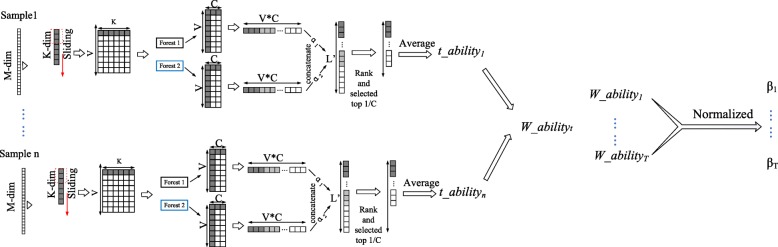
The basic structure of the sorting optimization algorithm. Different weights are assigned to the class vectors generated by different sliding windows

As using different sliding windows to extract class vectors greatly influences the final classification results, different weights are assigned to the class vectors generated by different sliding windows; this algorithm is called the sorting optimization algorithm (as shown in Fig. 3). The basic structure of the algorithm is as follows.

1. Suppose the number of samples is *N*, the size of the original features is *M*, and the number of class labels for the sample is *C*. For each sample i, we use a sliding window t to cut features (the index of the current window is t (1≤ t ≤ T). The size of the sliding window is *L* (1≤ L ≤ M). The stride of the scan is *S* (default S =1). We designate the number of feature vectors after scanning as *V* and calculate this value with formula (7).

7 $$ V=\frac{M-L}{S}+1 $$

(2) Each *L*-dim feature vector is input into one random forest and one completely random forest. The two forests each output a *C*-dim class vector. Each random forest uses *V L*-dim feature vectors as input and outputs *V C*-dim class vectors. Then, these *V C*-dim class vectors are concatenated into V * *C*-dim class vectors (called *RF-vec*). Similarly, we concatenate the class vectors from the output of the completely random forest and call it c*RF-vec*.
(3) *RF-vec* and c*RF-vec* are multiplied by their respective weights α_1_ and α_2_ (the weights of the different random forests, calculated in the previous section) and concatenated as 2 ∗*V* ∗ *C*-dim class vectors (length *L*^′^ = 2 ∗ *V* ∗ *C*).
(4) First, the *L*^′^-dim class vector obtained in step (3) is sorted in descending order. Then, the average of the top 1/*C* of the sorted class vector values is calculated. This indicator can be viewed as the approximate ability of the current window to predict the current sample *i*.We name it *t* _ *ability*_*i*_, and it is calculated as shown in formula (8).

8 $$ t\_ abilit{y}_i\;\frac{\sum_{j=1}^{\frac{L^{\hbox{'}}}{C}} Desc\left( conc\left( RF- vec\ast {\alpha}_1, cRF- vec\ast {\alpha}_2\right)\right)}{\frac{L^{\hbox{'}}}{C}} $$

where *conc* represents the concatenate operation.

(5) Steps (1)–(4) are repeated for the *N* samples, and we obtain the prediction abilities for the current window for the *N* samples (*t* _ *ability*_1_, *t* _ *ability*_2_, …, *t* _ *ability*_*N*_). This indicator approximates the prediction performance of the current window *t*.
(6) The prediction ability *W* _ *ability*_*t*_ of the sliding window *t* is obtained by averaging the prediction abilities of the current window for the *N* samples, as shown in formula (9).

9 $$ W\_{ability}_t=\frac{\sum \limits_{i=1}^nt\_{ability}_i}{n} $$

(7) Steps (1)–(6) are repeated to obtain the prediction ability of each window (*W* _ *ability*_1_…*W* _ *ability*_*t*_…*W* _ *ability*_*T*_). The individual *W* _ *ability* values are normalized to obtain the predictive weights *β*_*t*_ of each sliding window, as shown in formula (10), which is used to obtain the weights of each window *β*_1_ *β*_2_ … *β*_*t*_ … *β*_*T*_.

10 $$ {\upbeta}_t=\frac{W\_{ability}_t}{\sum \limits_{t=1}^TW\_{ability}_t} $$

The class vector obtained from each window is multi-plied by its corresponding β value and then concatenated with each other as the output of the multi-grained scanning component.

In step (4) of the algorithm, we calculate the average of the top 1/*C* class vector values to approximate the current scan window prediction ability because the random forest outputs the confidence probabilities that the samples belong to a certain class. If the maximum value of the confidence probabilities is closer to 1, the random forest has a stronger ability to distinguish the sample categories. Therefore, we take the average of the top 1/*C* class vector values to approximate the current scan window prediction ability.

After assigning various weights to the gcForest algorithm, we obtain the MLW-gcForest classification model.

### MLW-gcForest decision fusion of multi-modal data

The pathogenesis of lung adenocarcinoma is complex, and satisfactory staging results are often difficult to obtain using only single-modal data. A more accurate diagnosis of lung adenocarcinoma is achieved by combining multi-modal data (methylation data, RNA-seq data and CNV data) and taking full advantage of the complementarity between the advanced features of the different modal datasets. Therefore, multi-modal lung adenocarcinoma genetic data are used to train different MLW-gcForest models, and decision-level fusion is performed.

The basic idea of decision-level fusion is to determine which class a sample belongs to by considering the classification results of multiple models. In our algorithm, we obtain the final classification results via weighted voting of multiple models.

Each classification models *h*_*p*_ predicts a label from the category label set {*class*_1_, *class*_2_, …, *class*_*C*_}. The forecast output is represented as a *C*-dim class vector $\left({h}_p^1\left(\mathrm{x}\right);{h}_p^2\left(\mathrm{x}\right);\dots {h}_p^C\left(\mathrm{x}\right)\right)$, such as (0.12, 0.33, …, 0.45), where ${h}_p^q\left(\mathrm{x}\right)$ is the output of *h*_*p*_ on *class*_*q*_. Different types of individual models can produce different types of ${h}_p^q\left(\mathrm{x}\right)$ values (${h}_p^q\left(\mathrm{x}\right)\in \left[0,1\right]$). The weighted voting method used in this paper is shown in the following formula:

11 $$ H(x)={class}_{argmax_q}{\sum}_{p=1}^m{\gamma}_p{h}_p^q(x) $$

*m* is the number of modalities of the data, and *γ*_*p*_ is the degree of influence of modality *p* on the classification results based on the experimental results, where *γ*_*Methylation*_ + *γ*_*RNA*_ + *γ*_*CNV*_ = 1. *γ*_*Methylation*_, *γ*_*RNA*_, and *γ*_*CNV*_ are normalized by the accuracies of each type of data’s MLW-gcForest model, as shown in formula (12).

12 $$ {\gamma}_p=\frac{acc_{m_p}}{\sum_{i=1}^m{acc}_{m_i}} $$

*m*_*p*_ is the model trained by modality *p*.

**Fig. 4:**
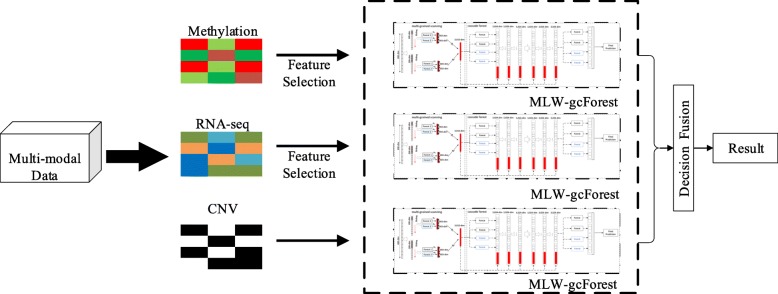
MLW-gcForest decision fusion of multi-modal data. Multi-modal (gene expression RNA-seq, methylation data and CNV) lung adenocarcinoma genetic data are used to train different MLW-gcForest models, and decision-level fusion is performed

Finally, weighted voting is performed on the MLW-gcForest model trained using three different modal datasets to obtain the final classification result (as shown in Fig. 4).

## Results

### Materials

To evaluate the performance of the MLW-gcForest algorithm, methylation data, RNA-seq data, CNV data and corresponding clinical data of lung adenocarcinoma are downloaded from the TCGA [37] (https://portal.gdc.cancer.gov/). The data include 492 methylation samples, 576 RNA-seq samples, and 516 CNV samples. After excluding samples without clinical staging and feature values that were missing more than 50% of information, we obtained 155 cases of stage I data, 243 cases of stage II data, 41 cases of stage III data, and 16 cases of stage IV data. The small number of samples in stage IV (less than 20 samples) would result in a very unbalanced training sample; therefore, the data for stage IV were excluded. Then, we excluded samples that did not have complete multi-modal data and obtained 369 samples of multi-modal data as our final dataset.

Each sample has 485,577 columns of feature values for the methylation data, 60,483 columns of feature values for the RNA-seq data, and 39 columns of feature values for the CNV data (all corresponding to valid data columns after null values are deleted). After feature selection, the methylation data retained 340 columns, RNA-seq retained 320 columns and CNV had too few columns to perform feature selection.

### Experiment

**Fig. 5:**
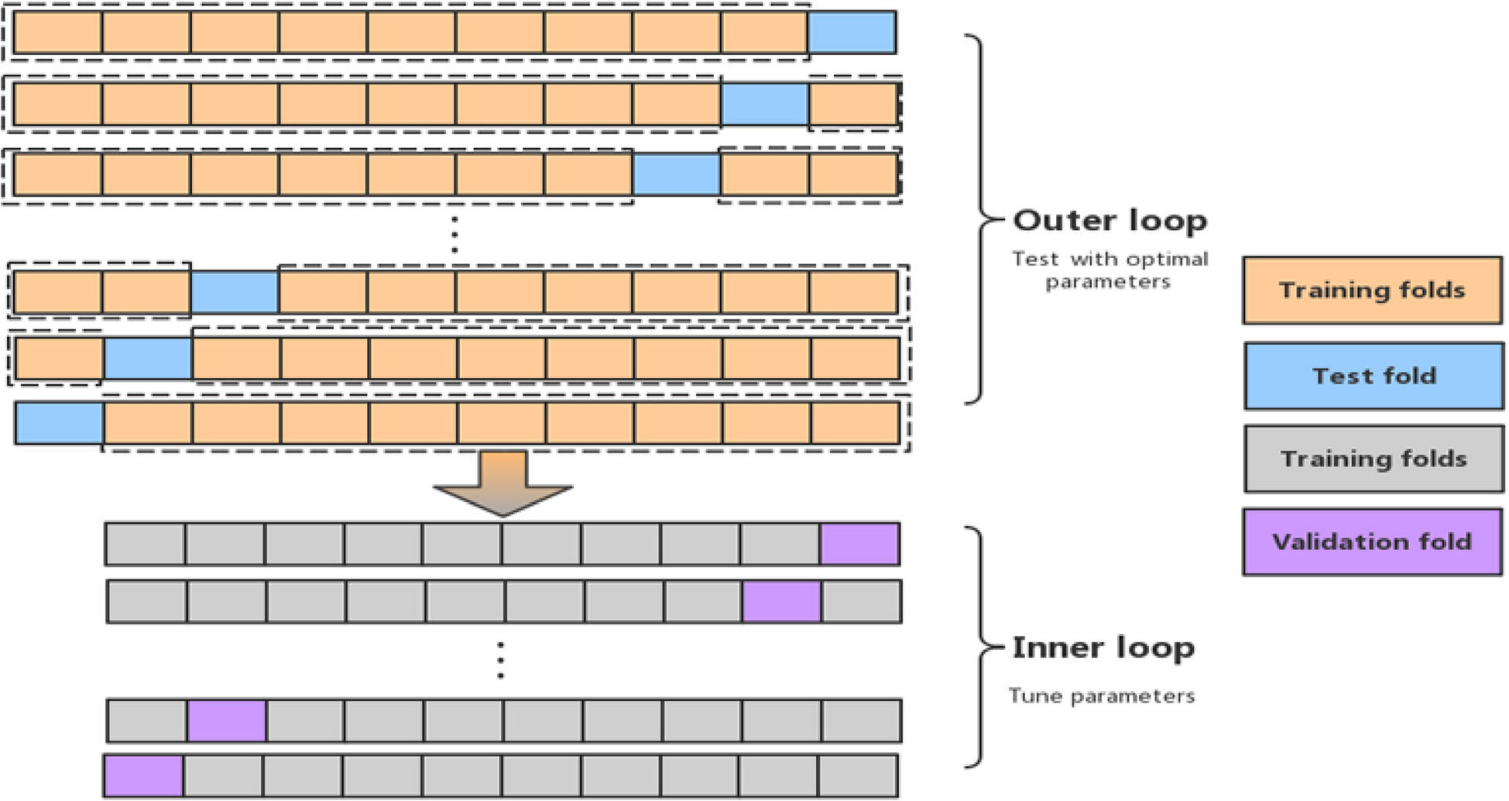
Nested cross-validation process, which includes an outer loop and an inner loop. The inner cross-validation loop is used to perform parameter adjustments, while the outer cross-validation loop is used to calculate the final error estimate for model performance

To evaluate the performance of the proposed algorithm, we use nested cross-validation [38] to train and test the model. Compared with standard cross-validation, nested cross-validation can achieve an almost unbiased estimation of model performance [38]. The process of nested cross-validation is divided into an outer loop and an inner loop, as shown in Fig. 5. The inner loop is used to perform parameter adjustments, while the outer loop is used to calculate the final error estimate for model performance.

As shown in Fig. 5, we divide the dataset into ten folds in a mutually exclusive manner. Each time we select nine folds to execute the inner loop (the inner loop performs standard 10-fold cross-validation), and the remaining fold is used for testing. The above process is the outer loop. The outer loop is repeated 10 times until each fold is used as a test set. Therefore, results are obtained for 10 test sets. We calculated the average of the ten test results from the nested 10-fold cross-validation.

It is worth noting that the inner loop performs standard 10-fold cross-validation; that is, the data used to execute the inner loop are divided into ten folds, nine folds are selected for training, and the other fold is used for validation. This process is repeated 10 times until each fold is used as the validation set. Moreover, when performing the inner loops, if the error on the training data continues to decrease but the error on the validation data stops decreasing, the training process terminates early, even before reaching the maximum number of epochs to avoid overfitting.

As described in section 2.4, we trained different MLW-gcForest models using multi-modal data with nested 10-fold cross-validation and then performed decision-level fusion.

In our algorithm, one random forest and one completely random forest were set up in the multi-grained scanning, and 300 decision trees were used in each forest. In the cascade forest layer, we used 300 decision trees for two completely random forests and two random forests. For better comparison with existing algorithms, we used different machine learning algorithms to construct lung adenocarcinoma staging models based on single and multi-modal data (methylation, RNA, CNV): SVM, K-nearest neighbors (KNN), logistic regression (LR), random forest (RF), gcForest and the proposed MLW-gcForest. We considered commonly used evaluation indexes, namely, AUC, accuracy, precision, recall and F_1_ score, to evaluate the performance of the algorithm.

### Result of lung adenocarcinoma staging models based on single-modal data

**Fig. 6:**
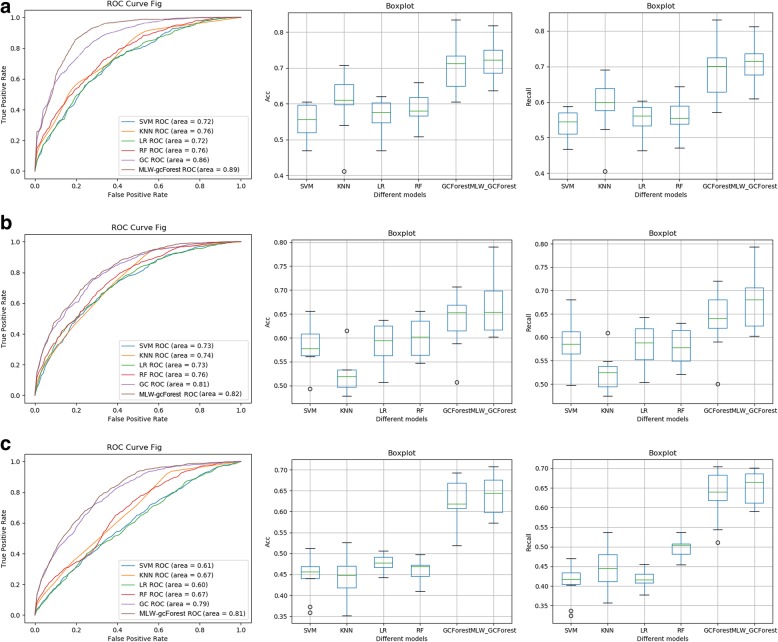
The results from the lung adenocarcinoma staging model based on different single-modal datasets with different algorithms. Row (**a**) contains the results from the use of the methylation data with different algorithms: (a1) ROC curves; (a2) Accuracy; (a3) Recall. Row (**b**) contains the results from the use of the RNA-seq data with different algorithms: (b1) ROC curves; (a2) Accuracy; (a3) Recall. Row (**c**) contains the results from the use of the CNV data with different algorithms: (c1) ROC curves; (c2) Accuracy; (c3) Recall

To evaluate the performance of the MLW-gcForest algorithm, MLW-gcForest, gcForest, and traditional machine learning methods were used to build lung adenocarcinoma staging models from different single-modal data. Based on the different single-modal datasets, we trained different methods with nested 10-fold cross-validation to evaluate the classification ability (Fig. 6). The three rows of Fig. 6 show the classification performance of different algorithms based on methylation, RNA, and CNV measured through nested 10-fold cross-validation. The three columns in Fig. 6 show the classification performance of different algorithms based on different modal data under the same evaluation metric. As shown in Fig. 6a(1), b(1) and c(1), the AUC values of the proposed MLW-gcForest algorithm are higher than those of the remaining algorithms on all three modal datasets.

The accuracy of the different algorithms on each data modality is shown in Fig. 6a (2), b(2), and c(2). The accuracy of the MLW-gcForest algorithm is higher than that of gcForest, SVM, KNN, LR and RF.

In addition, we also compare the precision and F_1_ scores of MLW-gcForest with those of the other algorithms in Table 1. Table 1 shows that our algorithm achieves a precision of 0.771 and an F_1_ score of 0.767, which are higher than those of the standard gcForest (precision and F_1_ score of 0.715 and 0.709) in methylation data. MLW-gcForest always outperforms gcForest, and their performances are superior to those of traditional machine methods on the other two single-modal data (RNA and CNV). This result demonstrates that the deep forest structure can capture more complex and diverse features, making it more suitable for small-sample genetic data. Furthermore, the proposed multi-level weighting strategy can help deep forests extract more valuable multi-level features, thus effectively improving the classification ability of the standard gcForest on small-sample genetic data.

**Table 1:** Performance comparison of different modal data with different methods

Classification algorithm	Methylation		RNA		CNV	
	Precision	F_1_	Precision	F_1_	Precision	F_1_
SVM	0.524	0.519	0.552	0.558	0.427	0.434
KNN	0.584	0.605	0.533	0.528	0.460	0.466
LR	0.575	0.572	0.609	0.603	0.446	0.486
RF	0.606	0.618	0.611	0.602	0.512	0.557
gcForest	0.715	0.709	0.634	0.643	0.616	0.628
MLW-gcForest	0.771	0.767	0.659	0.669	0.675	0.677

### Comparison of the staging of lung adenocarcinoma based on different algorithms using multi-modal data

**Fig. 7:**
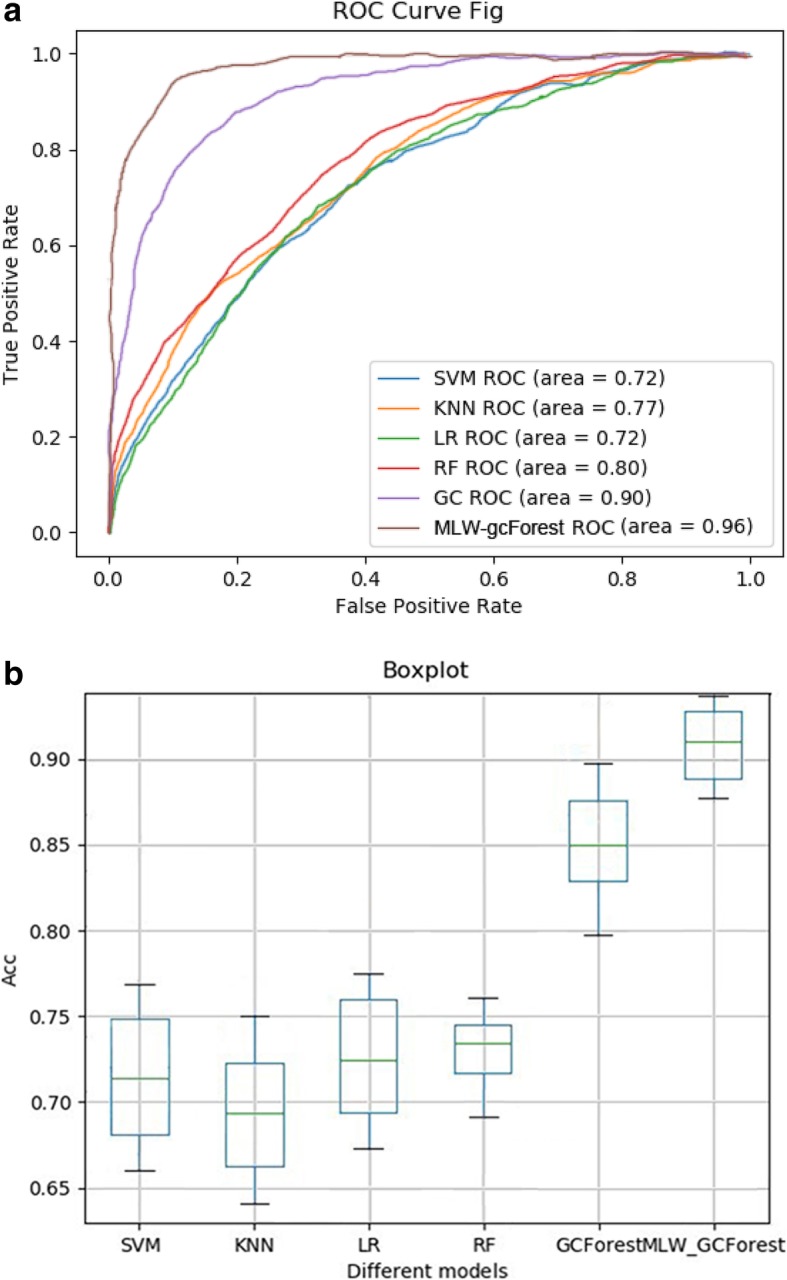
Performance comparison using different algorithms based on multi-modal data: (**a**) ROC curve, (**b**) Accuracy

We used different classification algorithms to construct staging models of lung adenocarcinoma based on multi-modal data to demonstrate the performance of the proposed MLW-gcForest algorithm in integrating multi-modal data for lung adenocarcinoma staging. In addition to plotting the ROC curves and calculating the AUC, the accuracy for each method under the multi-modal data measured through nested 10-fold cross-validation was calculated and is shown in Fig. 7. As shown in Fig. 7, the MLW-gcForest algorithm achieves better classification results on the staging of lung adenocarcinoma. The AUC and accuracy values of MLW-gcForest are higher than those of the standard gcForest algorithm and traditional machine learning algorithms.

The precision, recall and F_1_ score of the proposed MLW-gcForest and the other algorithms are shown in Table 2. The precision, recall and F_1_ score of the MLW-gcForest classification reached 0.896, 0.882 and 0.889, respectively, which are higher than those of the standard gcForest (0.764, 0.795, and 0.779). The comparison results indicate that MLW-gcForest and gcForest perform significantly better than the other traditional machine learning algorithms when using multi-modal data for lung adenocarcinoma staging. The results suggest that the deep forest algorithms (MLW-gcForest and gcForest) are more effective in lung adenocarcinoma staging because more complex and diverse features can be captured to distinguish different classes.

**Table 2:** The effects of different classification algorithms on the precision, recall, and F_1_ score of the staged model of the multi-modal data

Algorithm	Precision	Recall	F_1_
SVM	0.674	0.664	0.669
KNN	0.664	0.646	0.655
LR	0.675	0.669	0.672
RF	0.706	0.730	0.718
gcForest	0.764	0.795	0.779
MLW-gcForest	0.896	0.882	0.889

Furthermore, our result indicates that comprehensive multi-modal genetic data and the multiple decision-level fusion strategies of the MLW-gcForest model effectively improve the accuracy of lung cancer staging because the proposed algorithm not only enables the deep forests to extract more valuable and multi-level features through improved multi-level weighting strategies but also effectively utilizes the complementarity of multi-modal genetic data.

### Comparison of multi-modal data and single-modal data in the staging of lung adenocarcinoma with the MLW-gcForest algorithm

**Fig. 8:**
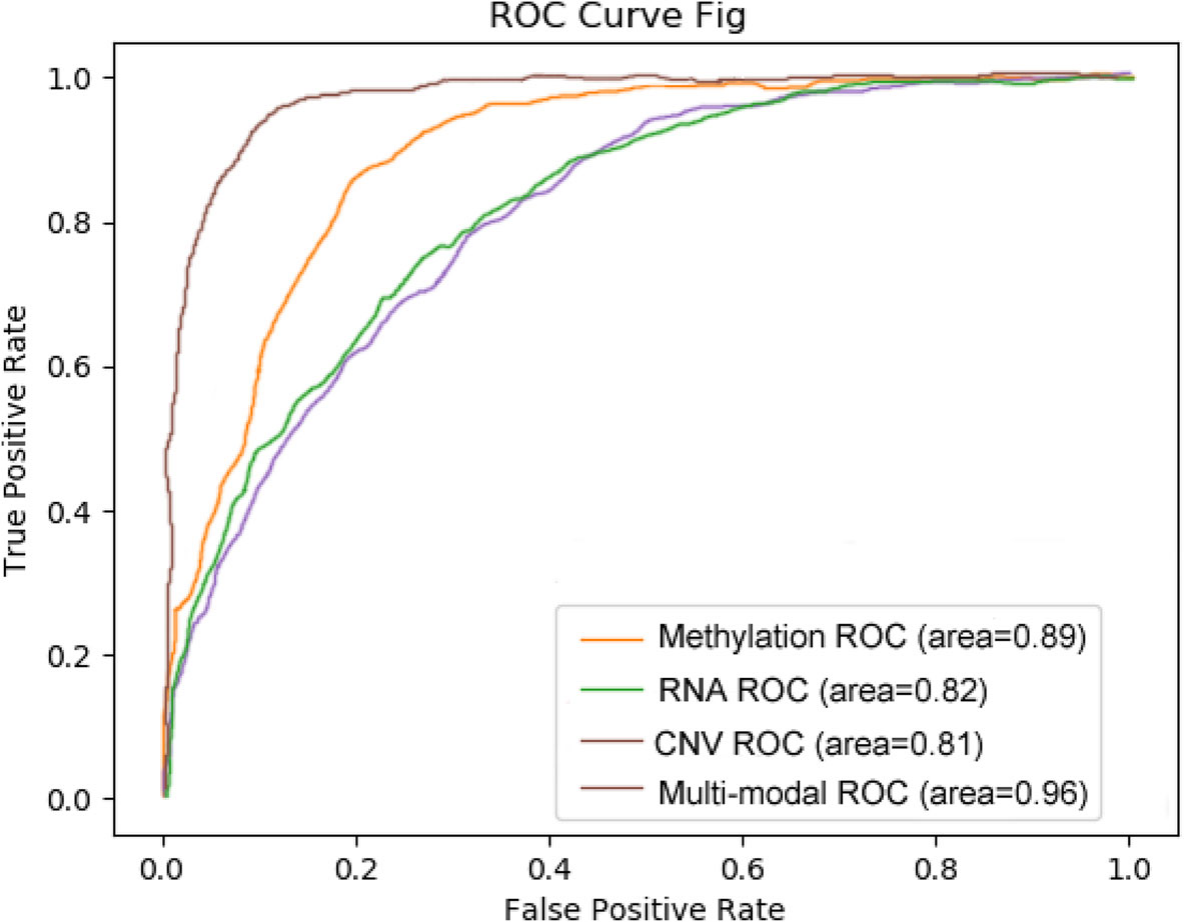
Performance comparison between multi-modal, methylation, RNA and CNV

To confirm the effectiveness of the multi-modal data, we compared the classification performance of MLW-gcForest using single-modal (methylation, RNA-seq, CNV) and multi-modal data. We plotted the ROC curves and calculated the AUC value between different modal data measured through nested 10-fold cross-validation, as shown in Fig. 8. The results indicate that the MLW-gcForest algorithm achieves better classification performance when using multi-modal data than when using single-modal data. In addition, comparing the accuracy in the multi-modal scenario in Fig. 7b with that in the single-modal scenario in Fig. 6a(2), b(2) and c(2), it can be seen that the integration of multi-modal data effectively improves the accuracy of lung adenocarcinoma stage prediction. Table 3 shows the accuracy, precision, recall, and F_1_ score of the lung adenocarcinoma staging model measured through nested 10-fold cross-validation of different modal datasets. The results indicate that MLW-gcForest achieves better performance (accuracy 0.908, precision 0.896, recall 0.882, F_1_ 0.889) with multi-modal data than with single-modal data. Using multi-modal data in the proposed MLW-gcForest significantly improved the accuracy of lung adenocarcinoma staging, which suggests that integrating multi-modal genetic data can effectively improve the accuracy of lung adenocarcinoma staging compared to using only single-modal data.

**Table 3:** Performance of the lung adenocarcinoma staging model with different modalities of data

Modality	Accuracy	Precision	Recall	F_1_
Methylation	0.751	0.771	0.763	0.767
RNA	0.689	0.659	0.679	0.669
CNV	0.645	0.675	0.677	0.677
Multi-modal	0.908	0.896	0.882	0.889

Table 3 also shows that the methylation data have a higher staging classification ability for lung adenocarcinoma than do RNA-seq and CNV data.

## Discussion

Methylation data are found to be the most discriminative in building lung adenocarcinoma staging models using single-modal data. MLW-gcForest based on multi-modal genetic data achieves better classification performance than that with single-modal data. These results indicate that combining multi-modal genetic data is an efficient way to improve the classification ability of lung adenocarcinoma staging. We also found that in the process of staging lung adenocarcinoma, the MLW-gcForest model and the gcForest model are superior to the traditional machine learning algorithms. The most likely reason is that deep forests (MLW-gcForest model, gcForest) can learn more valuable and advanced features with multi-grained and cascade layers. In addition, MLW-gcForest outperformed the standard gcForest on most lung adenocarcinoma datasets. The results suggest that our multi-level weighting strategy effectively improves the classification ability of the standard gcForest model on small-sample cancer datasets.

**Fig. 9:**
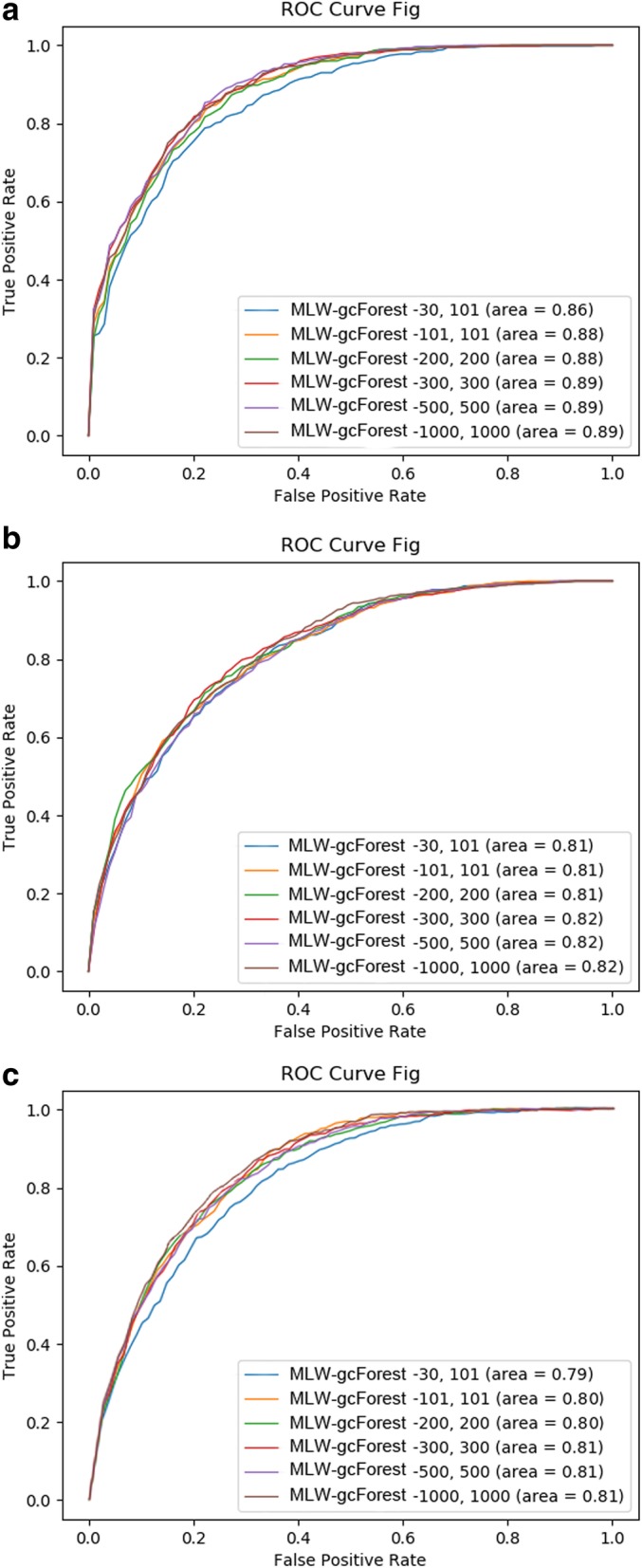
Comparison of MLW-gcForest models constructed with different numbers of decision trees in the decision forests for (**a**) methylation data, (**b**) RNA-seq data, and (**c**) CNV data

Now, we explain why 300 decision trees are chosen in our algorithm. We conducted comparative experiments to determine the required number of decision trees in the RF for the algorithm to achieve the best results. Figure 9 shows the results of these comparative experiments.

As shown in Fig. 9, changing the number of decision trees has little effect on the results when using RNA-seq data (Fig. 9b) but a larger effect on the results when using methylation (Fig. 9a) and CNV (Fig. 9c) data. For the methylation and CNV data, the worst performance is obtained when the number of decision trees in the algorithm is set to [30, 101] (i.e., the number of decision trees in the RF at the multi-grained scanning step is set to 30 and that of the cascade forest is set to 101). The algorithm performs best when the number of trees is set to [300, 300] or [500, 500].

Based on the above comparative results, [300, 300] was selected as the final experimental parameters: although [300, 300] and [500, 500] yielded similar experimental results, the time and calculation costs of using [300, 300] are lower.

Other scholars have used machine learning algorithms to stage lung adenocarcinoma. Li et al. [6] provided a staging model of lung cancer with an accuracy of 0.71. Nicolas Anthony Nguyen et al. [7] used SVM to classify TNM staging in lung cancer patients, with overall accuracies of 0.64 and 0.82 across T and N stages, respectively. The comparison of the experimental results shows that our proposed algorithm achieves higher AUC values and accuracy for lung adenocarcinoma classification. Moreover, MLW-gcForest based on multi-modal genetic data is an effective method to improve the classification model of lung adenocarcinoma compared to traditional machine learning algorithms.

## Conclusion

In this paper, we propose an improved gcForest model called MLW-gcForest and implement decision-level fusion to address the challenge of staging lung adenocarcinoma using small-sample multi-modal genetic data. The experimental results show that the MLW-gcForest algorithm has an AUC of 0.96 and an accuracy of 0.908 for lung adenocarcinoma staging, which are better than those achieved by the standard gcForest and traditional machine learning algorithms. Therefore, the proposed MLW-gcForest algorithm is more suitable for small-sample genetic data, and the integration of multi-modal genetic data can effectively improve the accuracy of lung adenocarcinoma staging compared to that achieved with single-modal data.

Although the experimental results show that the proposed combination of MLW-gcForest and multi-modal genetic data has the potential to improve the staging of lung adenocarcinoma, some limitations remain in our research. First, the amount of multi-modal genetic data collected in the experiment is relatively small, which may limit the training of more powerful lung adenocarcinoma staging models. Second, our experiments integrate only three types of genetic data, namely, methylation data, RNA-seq, and CNV, which may ignore the value of other types of genetic data for the staging of lung adenocarcinoma. In addition, pathological images, another class of valuable data found in the TCGA, are not considered in our study. In our future work, we will collect more types of genetic data to train and test the proposed model and explore the possibility of combining pathological images with multi-modal genetic data for the staging of lung adenocarcinoma. In addition, we intend to extend the proposed algorithm to the classification task of cancer subtypes.

## Data Availability

The datasets used and/or analyzed during the current study are available from the corresponding author on reasonable request.

## Abbreviations

AUC: Area under the curve
CNV: Copy number variation
Dim: Dimensional
HUM: Hypervolume under multi-flow
KNN: K-nearest neighbors
LR: Logistic regression
MLW-gcForest: Multi-weighted gcForest
NSCLC: Non-small cell lung cancer
RF: Random forest
ROC: Receiver operating characteristics
SVM: Support vector machine
TCGA: The Cancer Genome Atlas
TNM: Tumor node metastasis
